# A Practical Approach for Predicting Antimicrobial Phenotype Resistance in *Staphylococcus aureus* Through Machine Learning Analysis of Genome Data

**DOI:** 10.3389/fmicb.2022.841289

**Published:** 2022-03-02

**Authors:** Shuyi Wang, Chunjiang Zhao, Yuyao Yin, Fengning Chen, Hongbin Chen, Hui Wang

**Affiliations:** ^1^Institute of Medical Technology, Peking University Health Science Center, Beijing, China; ^2^Department of Clinical Laboratory, Peking University People’s Hospital, Beijing, China

**Keywords:** *Staphylococcus aureus*, k-mer algorithm, antimicrobial resistance (AMR), machine learning, WGS

## Abstract

With the reduction in sequencing price and acceleration of sequencing speed, it is particularly important to directly link the genotype and phenotype of bacteria. Here, we firstly predicted the minimum inhibitory concentrations of ten antimicrobial agents for *Staphylococcus aureus* using 466 isolates by directly extracting k-mer from whole genome sequencing data combined with three machine learning algorithms: random forest, support vector machine, and XGBoost. Considering one two-fold dilution, the essential agreement and the category agreement could reach >85% and >90% for most antimicrobial agents. For clindamycin, cefoxitin and trimethoprim-sulfamethoxazole, the essential agreement and the category agreement could reach >91% and >93%, providing important information for clinical treatment. The successful prediction of cefoxitin resistance showed that the model could identify methicillin-resistant *S. aureus*. The results suggest that small datasets available in large hospitals could bypass the existing basic research and known antimicrobial resistance genes and accurately predict the bacterial phenotype.

## Introduction

Traditional microbial identification and antimicrobial susceptibility rely on microbial culture technology, which is a time-consuming process. Owing to the optimization of the cultivation technology for more than a hundred years, the cultivation time has been shortened to within 24–48 h, depending on the specific strain. However, traditional culture techniques cannot meet the ever-increasing demands for rapid diagnosis. Although mass spectrometry technology has now been widely used, and it is possible to quickly identify pathogenic bacteria after obtaining purely cultured isolates, antimicrobial susceptibility results are still not widely available.

In recent years, gene sequencing technology has developed rapidly. The price of whole genome sequencing has fallen below 26.3 dollars a isolate recently, and the time spent on data yield can be archived within 18 h. Metagenomics technology can identify the types of pathogens in samples, including those which cannot be identified by traditional methods, within 8 h; thus expanding the boundaries of clinical microbiology and filling the gap between the genotype and phenotype of bacteria (Chen et al., 2020a,b).

After obtaining genome data, a relatively rough prediction of resistance can be achieved by studying the antimicrobial resistance genes in the genome. Nowadays, many studies have focused on antimicrobial resistance prediction; some studies only predicted whether isolates were resistant or susceptible, and did not predict the specific minimum inhibitory concentration (MIC) values (Brinda et al., 2020; Macesic et al., 2020; Avershina et al., 2021), while others predicted resistance and susceptibility based on the presence or absence of known resistance genes or single nucleotide polymorphisms. However, the updation of pre-existing basic research is quite slow (Satta et al., 2018; Khaledi et al., 2020; Kim et al., 2020; Avershina et al., 2021). The k-mer algorithm can count the size of genomic repeats and the degree of genomic heterozygosity. Currently, training set data used by k-mer based research is relatively large, while longer k-mers are better at exhibiting specificity of genomic features. This poses serious challenges to data acquisition, storage, and processing, thereby limiting the promotion and application of this technology. According to Moore’s Law (Moore, 1965), the speed of technological process double about every 2 years, revealing the speed of technological progress. It is foreseeable that these limitations will be overcome quickly in the future, and that follow-up developments will come rapidly, increasing the speed of the genome identification and analysis.

Methicillin-resistant *Staphylococcus aureus* (MRSA) is one of the most serious multi-antimicrobial-resistant threats (Tacconelli et al., 2018) and is the leading cause of many systemic infections (Lowy, 1998). In this study, we created a novel *Staphylococcus aureus* resistance prediction model that could accurately predict MICs of antimicrobial agents using a relatively small number of training isolates based on antimicrobial-resistant phenotypes and k-mer calculation, combined with machine learning algorithms.

## Materials and Methods

### Isolates

We prospectively investigated the pathogen spectrum of bloodstream infections, hospital-acquired pneumonia, and intra-abdominal infections. We collected 466 *S. aureus* [249 MRSA and 217 methicillin-susceptible *Staphylococcus aureus* (MSSA)], belonging to 23 different sequence types (STs), from 14 states across China between 2005 and 2020.

### Whole Genome Sequencing

The *S. aureus* isolates were sequenced using the Illumina NextSeq 500, NovaSeq, Hiseq Xten, and Illumina HiSeq platforms. Multilocus sequence typing (MLST) was performed according to the PubMLST scheme.^1^ We obtained clean DNA sequences after using the fastp program^2^ (Lee et al., 2018) to optimize raw FASTQ data quality and clean the raw data obtained after whole genome sequencing. Staphylococcal cassette chromosome mec (SCCmec) was assigned using SCCmecFinder-1.2 (De Oliveira et al., 2020)^3^ (Supplementary Table 1).

### Antibiotic Susceptibility Testing

Antibiotic susceptibility testing was performed on all the *S. aureus* isolates. The following antimicrobial agents were evaluated in this study: clindamycin (CLI), cefoxitin (FOX), oxacillin (OXA), levofloxacin (LVX), trimethoprim-sulfamethoxazole (SXT), vancomycin (VAN), linezolid (LNZ), erythromycin (ERY), daptomycin (DAP), and gentamicin (GEN). The MIC of commonly used antimicrobials was determined using the agar dilution method according to the protocol and the susceptibility spot of the Clinical and Laboratory Standards Institute guidelines (M100-S31,2021) and susceptible (S), intermediate (I) and resistant (R) categories were adjudicated.

### Data Set Preparation

For all isolates, the KMC application (Deorowicz et al., 2015)^4^ was used to extract features from cleaned DNA sequences. We used pandas (version 1.0.5) to combine and resize the obtained k-mer data, which were then combined into the matrix, with the rows indicating strain names and columns indicating k-mer counts. This matrix was used as the input data for machine learning (*k* = 11). The larger the value of *k*, the better the features obtained from the genomic information. In this study, *k* = 11 was used because of the limitations of computing memory. We used LabelBinarizer (scikit-learn, version 0.23.1) to convert the MIC labels to one-hot codes, which facilitated subsequent training of the model.

A total of 466 isolates with their antimicrobial susceptibility data were randomly divided into training sets (372 isolates) and testing sets (94 isolates). For GEN, ERY, and DAP, the data set was extracted separately because of the lack of antimicrobial susceptibility data. Among the 466 isolates, 69 isolates had GEN antimicrobial susceptibility data, 431 isolates had DAP antimicrobial susceptibility data, 454 isolates had ERY antimicrobial susceptibility data, and 20% were selected as the testing set. In order to ensure that all MICs of all antimicrobial agents could be trained as a category in the training set, we used the StratifiedShuffleSplit software (scikit-learn, version 0.23.1) to obtain stratified randomized folds. The folds were made by preserving the percentage of samples for each MIC grade. MIC grades that had only one isolate were incorporated into a lower grade for optimal classification.

### Machine Learning Analysis

The support vector machine (SVM) and random forest, used for training in Python 3.6, were based on scikit-learn (version 0.23.1) (Pedregosa et al., 2011). XGBoost is a machine learning algorithm based on the gradient boosting framework. We used the XGBoost (Chen and Guestrin, 2016) sklearn interface for training. We trained random forests with 600 trees. For SVM, we tried three kernel functions: linear, poly, and rbf. For XGBoost, we tried binary: logistic learning task parameters. For all training, we selected the best result of the 10-fold cross-validation as the final result. All source code is available at https://github.com/ShuyiWang-pku/sau_micprediction.

### Interpretation of Results

For the final classification, the results were evaluated according to the Clinical and Laboratory Standards Institute guidelines (M100-S31,2021). We assumed that for the categories obtained by the classifier, the classification results of one two-fold dilution, also termed as essential agreement (EA), were correct. According to this standard, the receiver operating characteristic (ROC) curves, area under curve (AUC) values, EA, category agreement (CA), sensitivity, specificity, negative predictive value (NPV), positive predictive value (PPV), very major error (VME), and major error (ME) were calculated to evaluate our model. ROC curves and AUC values can judge the predictive performance of models. Taking label imbalance into account, we calculated metrics globally by considering each element of the label indicator matrix as a label. We aggregated outcomes across all classes, drew the ROC curves and calculated the AUC values (Fawcett, 2006). CA refers to the accuracy of the prediction model that only considers the classification of susceptible, intermediate, and resistant categories of antimicrobial agents. Sensitivity refers to the number of predicted resistant and intermediate results divided by the actual number of resistant and intermediate samples, and specificity refers to the number of predicted susceptible results divided by the actual number of susceptible samples. NPV refers to the number of true negatives (a “susceptible” prediction for which the actual event had a “susceptible” result), divided by the total number of isolates predicted to be “susceptible” (sum of true negatives and false negatives). PPV refers to the number of true positives (a “resistant” or “intermediate” prediction, for which the actual result was “resistant” or “intermediate”), divided by the total number of isolates predicted to be “resistant” or “intermediate.” VME refers to the number of isolates predicted to be “susceptible” while the actual result was “intermediate” or “resistant,” divided by the actual number of “resistant” and “intermediate” results. ME refers to the number of isolates predicted to be “resistant” or “intermediate,” while their actual result was “susceptible” divided by the actual number of “susceptible” results.

## Results

### Basic Characteristics of the *Staphylococcus aureus* Isolates

**FIGURE 1:**
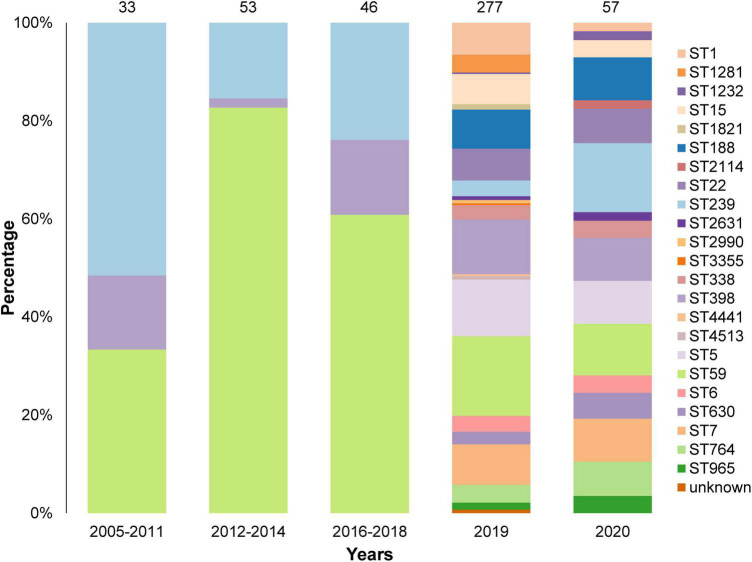
Sequence Types (STs) and collection years of the 466 isolates in this study. A total of 23 STs were identified and the STs of 2 isolates were unknown. Most isolates were collected in 2019 and 2020.

We analyzed 466 isolates of *S. aureus*, including 249 MRSA and 217 MSSA, from 14 states in China. The DNA sequence of all isolates was obtained using whole genome sequencing. Among the 466 *S. aureus* isolates, 23 STs were identified, among which ST59, ST239, and ST398 were higher in number (Figure 1). The ST of 2 isolates could not be identified; however, this could not be attributed to the quality of sequencing, as evidenced by the high quality sequencing data shown in the Supplementary Material. The sample collection details of all the isolates are presented in Table 1.

**TABLE 1:** States and collection years of the 466 isolates in this study.

Years States	2005–2011	2012–2014	2016–2018	2019	2020
Beijing	15	2	10	79	1
Chongqing	1	1	4	20	1
Guangdong	4	3	5	28	5
Hubei	2	-	4	19	-
Hunan	1	1	3	9	7
Jiangsu	1	2	3	22	-
Liaoning	1	1	-	-	-
Shaanxi	1	1	4	-	-
Shandong	1	38	1	20	6
Shanghai	4	1	2	5	6
Shanxi	-	-	3	-	25
Shenyang	-	2	2	-	-
Tianjin	-	-	2	26	-
Zhejiang	2	1	3	49	6

*The 466 isolates were widely distributed across 14 states in China.*

The MICs of the isolates are shown in Table 2, where light green refers to susceptible isolates defined according to clinical breakpoints, light orange refers to intermediate isolates, and colorless refers to resistant isolates. As seen from the results, VAN, LNZ, and DAP isolates were all susceptible. Therefore, we analyzed the classification performance of other models of antimicrobial agents and the factors that may affect the classification results.

**TABLE 2:** Number of genomes with different minimum inhibitory concentration (MIC) to the 10 antimicrobial agents for the 466 *Staphylococcus aureus* isolates used in this study.

MICs (μg/mL) Antimicrobial agents	0.032	0.064	0.125	0.25	0.5	1	2	4	8	16	32	64	128	256	512	Total
Clindamycin	46	163	32	3	5	1			3	1	2	2	208			466
Cefoxitin							46	171	31	54	63	7	94			466
Oxacillin					217	40	39	39	15	13	5	5	93			466
Levofloxacin			51	207	45	26	5	9	33	90						466
Trimethoprim-Sulfamethoxazole	102	257	43	21	8	10	9	2	2	12						466
Vancomycin					61	396	9									466
Linezolid					22	278	166									466
Erythromycin		4	33	112	6			4	9	6	9	12	12	10	237	454
Daptomycin			13	175	221	22										431
Gentamicin			8	34	3	5	1		1	4	2	1	5	3	2	69

*In this table, light green refers to susceptible isolates defined according to clinical breakpoints, light orange refers to intermediate isolates, and colorless refers to resistant isolates.*

### Extraction of the Characteristics of the Isolates and Rapid Identification of the *Staphylococcus aureus* Isolates by k-mer

**FIGURE 2:**
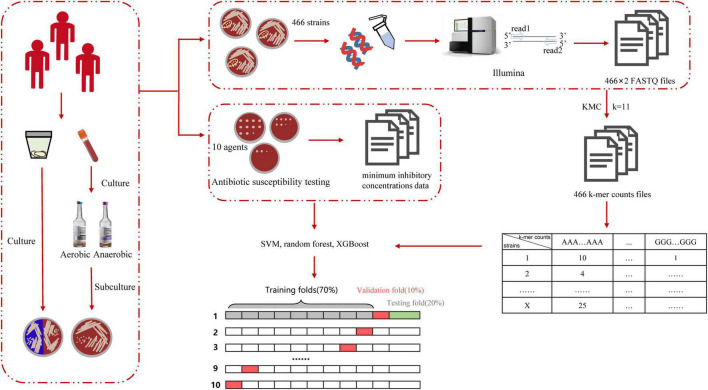
Schematic workflow of the actual operation in this study. DNA was isolated from the specimen and sequenced by Illumina to obtain the FASTQ file. We used KMC to obtain k-mer counts files and converted the format of the k-mer counts files to obtain the matrix, in which rows include isolates’ numbers and columns include k-mer counts. Finally, we used various machine learning algorithms to learn and obtain the prediction accuracy in the testing set.

**FIGURE 3:**
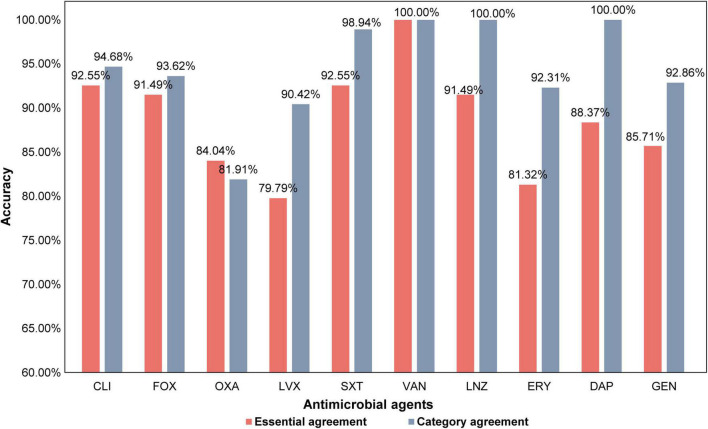
The prediction accuracy within the one two-fold dilution, essential agreement (EA), and the category agreement (CA) of all antimicrobial agents.

The workflow of this study is shown in Figure 2. After collecting and culturing the samples, we performed whole genome sequencing and antibiotic susceptibility testing for all isolates to obtain the genomic and antimicrobial susceptibility data, respectively. For our data, the k-mer algorithm was used to calculate the k-mer characteristics from the genome of each strain. The k-mer statistics (*k* = 11) of all isolates were combined into a matrix, which was used as the input data for machine learning. We used the following machine learning algorithms: SVM, random forest, and XGBoost (Chen and Guestrin, 2016) to train our data, and selected the best model to test the testing set, considered as the output results in this study. The testing time was consistently less than 1 min. The specific classification results for testing MIC and susceptible (S), intermediate (I), and resistant (R) categories are shown in Supplementary Tables 2–21, and the AUC, EA, CA, sensitivity, specificity, NPV, PPV, VME, and ME results are shown in Figure 3 and Table 3. To evaluate the reliability of the model, the ROC curves of five models with the best results in cross-validation were obtained (Supplementary Figures 1–10), and the average and standard deviations of the cross-validation results for all metrics are shown in Supplementary Tables 22, 23.

**TABLE 3:** The AUC (Area Under Curve), sensitivity, specificity, negative predictive value (NPV), positive predictive value (PPV), very major error (VME), and major error (ME) of the prediction results of all antimicrobial agents evaluated in this study.

Antimicrobial agents	AUC (%)	Sensitivity (%)	Specificity (%)	NPV (%)	PPV (%)	VME (%)	ME (%)
Clindamycin	94.61	91.30	97.92	92.16	97.67	8.70	2.08
Cefoxitin	92.65	94.00	93.18	93.18	94.00	6.00	6.82
Oxacillin	94.47	86.96	80.28	95.00	58.82	13.04	20.59
Levofloxacin	88.99	100.00	88.00	100.00	67.86	0.00	12.00
Trimethoprim-Sulfamethoxazole	92.77	100.00	98.92	100.00	50.00	0.00	1.08
Vancomycin	96.13	-	100.00	100.00	-	-	0.00
Linezolid	82.02	-	100.00	100.00	-	-	0.00
Erythromycin	94.83	96.49	85.29	93.55	91.67	3.51	14.71
Daptomycin	82.24	-	100.00	100.00	-	-	0.00
Gentamicin	85.46	100.00	91.67	100.00	66.67	0.00	8.33

The STs of the isolates in this study were different. In addition, we did not consider the evolution of the strain, unlike in a previous study (Brinda et al., 2020). Nonetheless, it was observed that the EAs and CAs of all antimicrobial agents were almost more than 80%. Except for OXA, LVX, and ERY, the EAs of all antimicrobial agents reached over 85%, while that for CLI and SXT reached over 92%. Except for OXA, the CAs for all antimicrobial agents reached over 90% (Figure 3). Furthermore, except for OXA, the sensitivity of all antimicrobial agents was over 90%, and the specificity of other antimicrobial agents was all over 85%. The sensitivity of LVX, SXT, and GEN reached 100%, and the specificity of CLI and SXT reached 97%. The VMEs of LVX, SXT, and GEN were as low as 0% in this study (Table 3).

### Effect of Data Volume and Structure on Prediction Results

For ERY and GEN, which had poor classification results, the specific data showed that the antimicrobial susceptibility data of these two antimicrobial agents were too biased toward the extreme value and the number of genomes in GEN was small, with only 69 isolates. When the MIC of GEN was 0.25 μg/mL, the number of isolates was 34, while the number of other MICs is relatively few, most of them are less than 5 (Table 1). When the MIC of ERY was 0.25 and 512 μg/mL, the number of isolates was 112 and 237, respectively, which was approximately 100–200 fold higher than that of some other MIC grades (Table 1).

We speculated that the data structure and data volume limit the improvement in accuracy. Owing to the difference in the number of samples, the classifier did not fully learn MIC features with a small number of isolates, which led to a vague distinction between the features of different categories and reduced the accuracy of model classification and recognition.

In most hospitals, data related to isolates is usually unevenly distributed, as observed in this study (Table 1). In addition to the fact that some large national institutions can use large hospital networks to obtain the data and avoid the condition where there only 1 or 2 isolates in some MIC, the distribution of antimicrobial susceptibility in most hospitals is not uniform, and the extremes of distribution phenomena are serious. This study proved that even if the antimicrobial susceptibility data of isolates are not evenly distributed, the developed model could be used to perform a certain rapid antimicrobial susceptibility analysis in various hospitals with a small amount of uneven data, and is suitable for most general hospitals that have accumulated a certain number of isolates with the popularity of sequencing technology. In addition, with an increase in the number of isolates collected in the future, the accuracy of MIC and classification may be further improved.

### Reasons for the Varied Prediction Results of Cefoxitin and Oxacillin

**FIGURE 4:**
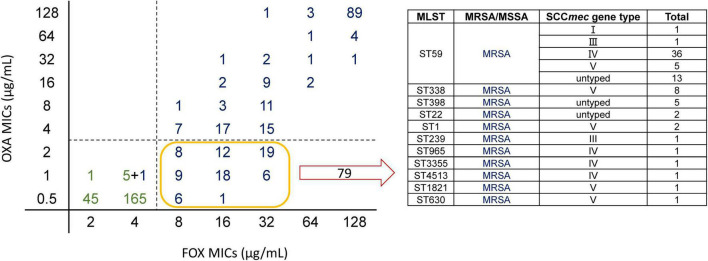
Scatter plot of MICs of cefoxitin (FOX) and oxacillin (OXA) and specific composition of OS-MRSA. On the left, the horizontal axis is the MICs of FOX for the isolates and the vertical axis is the MICs of OXA for the isolates. The figure depicts FOX MICs versus OXA MICs for *Staphylococcus aureus*. Green represents methicillin-susceptible *Staphylococcus aureus* (MSSA) and blue represents methicillin-resistant *Staphylococcus aureus* (MRSA) with Staphylococcal cassette chromosome *mec* (SCC*mec*) typing. Totally, 79 isolates were resistant to FOX and susceptible to OXA (OS-MRSA). The specific composition of these 79 isolates is shown in the table on the right; there were 56 ST59 isolates, accounting for the vast majority of OS-MRSA.

Methicillin, FOX, and OXA are β-lactam antimicrobials. In general, MRSA is resistant to OXA and FOX. This is because most MRSA isolates acquire the staphylococcal cassette chromosome *mec* (SCC*mec*) (Lakhundi and Zhang, 2018). However, in recent years, many studies have shown that even when carrying SCC*mec*, isolates are susceptible to OXA (termed as OS-MRSA). Previous studies have speculated that the development of OS-MRSA results from different SCC*mec* types, but there is no definitive conclusion (Boonsiri et al., 2020). Among the 466 isolates, 79 were resistant to FOX but susceptible to OXA. Among these 79 isolates, 56 were ST59, accounting for 70.89% of all OS-MRSA isolates (Figure 4). Among the ST59 isolates, OS-MRSA accounted for 46.67% of MRSA isolates (OS-MRSA/MRSA), which was almost half of all the ST59 MRSA isolates (Supplementary Table 24).

Our prediction model could directly capture the features of SCC*mec* from the FASTQ data and estimate the MICs of FOX, in which EA could reach 91.49% and CA could reach 93.62%. This result showed that the model could accurately and quickly distinguish whether the isolate was MRSA or MSSA. However, the prediction accuracy of OXA was relatively low, and CA was lower than that of EA because we considered one two-fold dilution for the calculation, while the MIC breakpoints of OXA were 2 and 4 μg/mL (Supplementary Table 24). The reason for the high accuracy of FOX may be that there is basically no “OS-MRSA” in FOX; that is, SCC*mec* can be detected; however, the isolate is susceptible to FOX. When the k-mer learns the genes of antimicrobial resistance, it is easy to distinguish them, but the isolates of ST59, which have SCC*mec* genes, were susceptible or resistant to OXA with a 50:50 proportion and have a close genetic relationship. Therefore, even if the classifier learns SCC*mec* genes, there will not be any clear conclusion, and the isolates may become OS-MRSA in different ways. We believe that the limited number of OS-MRSA interfered with the results, and our prediction model did not fully understand the characteristics of OS-MRSA, which resulted in varied prediction accuracies of OXA and FOX.

### Effect of Other Resistance Mechanism on Prediction Results

Previous studies have shown that ST59 is mostly susceptible to LVX and ST239 is mostly resistant to LVX (Li et al., 2018). Similar results were obtained in this study. Among the 133 ST59 isolates, only 11 isolates were intermediate or resistant to LVX, and the rest were all susceptible to LVX. Of the 54 ST239 isolates, only two were susceptible to LVX, and the rest were all intermediate or resistant. In contrast, ST59 and ST239 accounted for the majority of the total data set, and the two STs were very far apart at the genome level, and hence the CA of LVX was high. The low EA of LVX may be due to the small differences within susceptible isolates and resistant isolates, which our prediction model did not recognize.

### Timeliness of Prediction Using Genome Data

**FIGURE 5:**
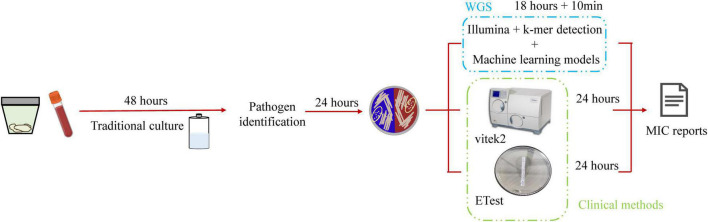
Schematic workflow of this study. Traditional microbial identification and antimicrobial agent susceptibility rely on microbial culture technology, which is time-consuming and requires 4 days to obtain the antimicrobial susceptibility report. This study used whole genome sequencing and was 6-h faster than routine clinical testing.

Antimicrobial resistance prediction was 6 h faster when using whole genome sequencing combined with k-mer detection and machine learning algorithms than the duration of routine clinical testing (Figure 5). The success of predicting MICs with pure bacterial culture with a limited amount of data, indicates that it is feasible to obtain susceptibility results directly from genomic data without requiring prior information.

## Discussion

In this study, the resistance characteristics of *S. aureus* to major antimicrobial agents were successfully predicted using k-mers and machine learning techniques. Among them, the CAs of all antimicrobial agents were generally high, and >90% pairs could provide important information for clinical treatment. Moreover, the EAs of most antimicrobial agents were higher than 85%.

Previous studies have predicted antimicrobial resistance phenotypes using antimicrobial-resistant genes or conserved genes in gene sequence of isolates (Kim et al., 2020; Macesic et al., 2020; Nguyen et al., 2020; Avershina et al., 2021), these methods of inferring antimicrobial resistance phenotypes from known resistance genes are highly accurate. At the same time, there are many public databases like PATRIC (Vanoeffelen et al., 2021), through which researchers can collect genomic data. The availability of such databases reduces the imbalance of data caused by regional differences by enabling the tracking of antimicrobial resistance evolution or prediction of antimicrobial resistance phenotype through artificial intelligence. The method reported in this study is an independent prediction method without information on resistance genes that can predict the resistance of isolates with unknown resistance mechanisms. This is the first time that k-mer has been combined with machine learning to predict the MIC of *S. aureus* in China. Most previous studies predicted antimicrobial resistance through known resistance mechanisms, and the resistance phenotypes have not been obtained directly from FASTQ files (Gordon et al., 2014; Mason et al., 2018; Wang et al., 2021). The findings of this study are consistent with those of previous studies.

*Staphylococcus aureus* is a common pathogenic gram-positive coccus, while former research objects have been mainly gram-negative bacteria (Nguyen et al., 2019; Van Camp et al., 2020) or *Mycobacterium tuberculosi*s (Satta et al., 2018). MRSA is more specific, and the resistance mechanism of MRSA corresponds to its resistant phenotype (Lee et al., 2018). However, the resistance mechanism of gram-negative bacteria is relatively complex; for example, *Enterobacteriaceae* can simultaneously develop resistance to many types of antimicrobials by producing different mechanisms, such as modifying enzymes, changing the action targets of antimicrobials, and reducing membrane permeability. Gram-negative bacteria often exhibit one resistance mechanism that causes resistance to multiple antimicrobial agents (De Oliveira et al., 2020). Therefore, in this study, for gram-positive bacteria, such as those with resistance mechanisms relatively consistent with the resistance phenotype, more accurate results can be obtained with a smaller number of training sets. For isolates with more complicated resistance mechanisms, such as gram-negative bacteria, more accurate prediction results need a larger number of training sets. The present study demonstrated the clinical value of this method in predicting the resistance phenotypes of gram-positive bacteria.

While a large number of training set isolates can greatly improve the accuracy of prediction results, it simultaneously limits the application scope of this method. For example, greater power is required for data computing and storage. As the *k* value increases, the size of the k-mer file increases by 4*^k^*-fold, which poses a considerable challenge to data processing. In addition, the large amount of training set data in the previous studies was not easily accessible, and most hospitals hardly obtained such clean data. In contrast, the sequencing data for less than 500 isolates in this study were more accessible. Almost all big hospitals can collect the same number of isolates in 1–2 years, and a strain prediction model based on its own regional characteristics can be rapidly established, thus enhancing its application. All samples used in this study are from one country, and our methodology focused on large hospital data, which may not be applicable to all regions because the genotypes are clustered geographically (Novembre et al., 2008). Collecting more samples from other countries or regions would solidify our approach.

In clinical practice, clinicians generally use antibiotics based on strain susceptibility results. The results from this study can help improve the accuracy of empirical treatment in the clinic, especially when there is no way to obtain antimicrobial susceptibility results quickly. In addition, extracting gene data characteristics using the k-mer alone can be linked with metagenomics. Analysis of the MIC directly after species determination can speed up pathogen diagnosis and antimicrobial susceptibility testing in the future.

## Data Availability Statement

The datasets presented in this study can be found in online repositories. The names of the repository/repositories and accession number(s) can be found in the article/Supplementary Material.

## Ethics Statement

The studies involving human participants were reviewed and approved by The Peking University People’s Hospital Institutional Review Board. Written informed consent for participation was not required for this study in accordance with the national legislation and the institutional requirements.

## Author Contributions

HW conceived, designed, and supervised the study. SW, CZ, YY, FC, and HC collected and interpreted the data. SW and CZ conducted the analysis. SW, CZ, and HW drafted the manuscript. All the authors approved the final version of the manuscript.

## Conflict of Interest

The authors declare that the research was conducted in the absence of any commercial or financial relationships that could be construed as a potential conflict of interest.

## Publisher’s Note

All claims expressed in this article are solely those of the authors and do not necessarily represent those of their affiliated organizations, or those of the publisher, the editors and the reviewers. Any product that may be evaluated in this article, or claim that may be made by its manufacturer, is not guaranteed or endorsed by the publisher.

## Footnotes

1
https://pubmlst.org/saureus/

2
https://github.com/OpenGene/fastp

3
https://cge.cbs.dtu.dk/services/SCCmecFinder-1.2/

4
https://github.com/lh3/kmer-cnt
