# Supplementary material for: A Practical Approach for Predicting Antimicrobial Phenotype Resistance in Staphylococcus aureus Through Machine Learning Analysis of Genome Data
Source: Front Microbiol. 2022 Mar 2;13:841289. doi: 10.3389/fmicb.2022.841289 (PMC8924536; doi:10.3389/fmicb.2022.841289)

A Practical Approach for Predicting Antimicrobial Phenotype Resistance in *Staphylococcus aureus* through Machine Learning Analysis of Genome Data

Shuyi Wang[1,2#], Chunjiang Zhao[2#], Yuyao Yin[2], Fengning Chen[1,2], Hongbin Chen[2], Hui Wang[1,2*]

[1]Institute of Medical Technology, Peking University Health Science Center, Beijing, China

[2]Department of Clinical Laboratory, Peking University People's Hospital, Beijing, China

[#]These authors contributed equally to this work.

Supplemental Materials

Table S2. Prediction of the minimum inhibitory concentration (MIC) for Clindamycin. Horizontal axis are the prediction results and the vertical axis are the standard results.

MICs (μg/mL)	0.032	0.064	0.125	0.25	0.5	8	128
0.032	1	7	0	0	0	0	1
0.064	6	26	0	0	0	0	1
0.125	1	3	2	0	0	0	1
0.25	0	0	0	0	0	0	1
0.5	1	0	0	0	0	0	0
8	0	0	0	0	0	0	1
128	1	0	0	0	0	0	41

Table S3. Prediction of the susceptible (S), intermediate (I) and resistant (R) categories for Clindamycin. Horizontal axis are the prediction results and the vertical axis are the standard results.

	S	R
S	47	4
R	1	42

Table S4. Prediction of the minimum inhibitory concentration (MIC) for Cefoxitin. Horizontal axis are the prediction results and the vertical axis are the standard results.

MICs (μg/mL)	2	4	8	16	32	64	128

2	4	5	0	0	0	0	1
4	4	28	0	0	0	0	2
8	0	0	2	4	0	0	0
16	1	0	2	1	7	0	0
32	2	0	0	3	7	0	1
64	0	0	0	0	1	0	0
128	0	0	0	0	1	1	17

Table S5. Prediction of the susceptible (S), intermediate (I) and resistant (R) categories for Cefoxitin. Horizontal axis are the prediction results and the vertical axis are the standard results.

	S	R
S	41	3
R	3	47

Table S6. Prediction of the minimum inhibitory concentration (MIC) for Oxacillin. Horizontal axis are the prediction results and the vertical axis are the standard results.

MICs (µg/mL)	0.5	1	2	4	8	16	32	64	128
0.5	45	2	0	0	0	0	0	0	1
1	4	1	1	0	0	0	0	0	0
2	2	2	0	2	0	0	0	0	0
4	4	0	3	2	0	0	0	0	0
8	1	2	1	0	0	0	0	0	0
16	2	0	0	1	0	0	0	0	0
32	1	0	0	0	0	0	0	0	0
64	0	0	0	0	0	0	0	0	1
128	0	0	0	0	0	0	0	0	16

Table S7. Prediction of the susceptible (S), intermediate (I) and resistant (R) categories for Oxacillin. Horizontal axis are the prediction results and the vertical axis are the standard results.

	S	R
S	57	3
R	14	20

Table S8. Prediction of the minimum inhibitory concentration (MIC) for Levofloxacin. Horizontal axis are the prediction results and the vertical axis are the standard results.

MICs (µg/mL)	0.125	0.25	0.5	1	2	4	8	16
0.125	4	5	0	1	0	0	0	0
0.25	2	40	0	0	0	0	0	0
0.5	4	5	0	0	0	0	0	0
1	4	0	0	1	0	0	0	0
2	0	0	0	0	0	0	0	1
4	1	0	0	0	0	0	1	0
8	2	1	1	0	0	0	1	2
16	2	2	0	0	0	0	1	13

Table S9. Prediction of the susceptible (S), intermediate (I) and resistant (R) categories for Levofloxacin. Horizontal axis are the prediction results and the vertical axis are the standard results.

	S	I	R
S	66	0	0
I	0	0	1
R	9	0	18

Table S10. Prediction of the minimum inhibitory concentration (MIC) for Trimethoprim-Sulfamethoxazole. Horizontal axis are the prediction results and the vertical axis are the standard results.

MICs (μg/mL)	0.032	0.064	0.125	0.25	0.5	1	2	16
0.032	14	7	0	0	0	0	0	0
0.064	12	35	5	0	0	0	0	0
0.125	0	4	5	0	0	0	0	0
0.25	3	0	1	0	0	0	0	0
0.5	2	0	0	0	0	0	0	0
1	0	0	0	0	1	0	1	0
2	0	0	1	0	0	0	1	0
16	0	1	0	0	0	0	0	1

Table S11. Prediction of the susceptible (S), intermediate (I) and resistant (R) categories for Trimethoprim-Sulfamethoxazole. Horizontal axis are the prediction results and the vertical axis are the standard results.

	S	R
S	92	0
R	1	1

Table S12. Prediction of the minimum inhibitory concentration (MIC) for Vancomycin. Horizontal axis are the prediction results and the vertical axis are the standard results.

MICs (μg/mL)	0.5	1	2
0.5	1	11	0
1	1	80	0
2	0	1	0

Table S13. Prediction of the susceptible (S), intermediate (I) and resistant (R) categories for Vancomycin. Horizontal axis are the prediction results and the vertical axis are the standard results.

	S
S	94

Table S14. Prediction of the minimum inhibitory concentration (MIC) for Linezolid. Horizontal axis are the prediction results and the vertical axis are the standard results.

MICs (μg/mL)	0.5	1	2
0.5	0	2	0
1	7	44	9
2	8	23	1

Table S15. Prediction of the susceptible (S), intermediate (I) and resistant (R) categories for Linezolid. Horizontal axis are the prediction results and the vertical axis are the standard results.

	S
S	94

Table S16. Prediction of the minimum inhibitory concentration (MIC) for Erythromycin. Horizontal axis are the prediction results and the vertical axis are the standard results.

MICs (μg/mL)	0.064	0.125	0.25	0.5	4	8	16	32	64	128	256	512
0.064	1	0	0	0	0	0	0	0	0	0	0	0
0.125	2	0	4	0	0	0	0	0	0	0	0	1
0.25	4	0	18	0	0	0	0	0	0	0	0	0
0.5	0	0	0	0	0	0	0	0	0	0	0	1
4	1	0	0	0	0	0	0	0	0	0	0	0
8	1	0	0	0	0	1	0	0	0	0	0	0
16	0	0	0	0	0	0	0	0	0	0	0	1
32	1	0	0	0	0	0	0	0	0	0	0	1
64	0	0	0	0	0	0	0	0	0	0	0	2
128	0	0	0	0	0	0	0	0	0	0	0	2
256	0	0	0	0	0	0	0	0	0	0	0	2
512	2	0	0	0	0	0	0	0	0	0	0	46

Table S17. Prediction of the susceptible (S), intermediate (I) and resistant (R) categories for Erythromycin. Horizontal axis are the prediction results and the vertical axis are the standard results.

	S	I	R
S	29	0	2
I	1	0	0
R	4	0	55

Table S18. Prediction of the minimum inhibitory concentration (MIC) for Daptomycin. Horizontal axis are the prediction results and the vertical axis are the standard results.

MICs (μg/mL)	0.125	0.25	0.5	1
0.125	2	1	2	0
0.25	8	18	9	0
0.5	6	8	28	0
1	0	2	2	0

Table S19. Prediction of the susceptible (S), intermediate (I) and resistant (R) categories for Daptomycin. Horizontal axis are the prediction results and the vertical axis are the standard

results.

	S
S	87

Table S20. Prediction of the minimum inhibitory concentration (MIC) for Gentamicin. Horizontal axis are the prediction results and the vertical axis are the standard results.

MICs (μg/mL)	0.125	0.25	0.5	1	16	32	128
0.125	2	0	0	0	0	0	0
0.25	1	6	0	0	0	0	0
0.5	0	1	0	0	0	0	0
1	1	0	0	0	0	0	0
16	1	0	0	0	0	0	0
32	0	0	0	0	0	0	1
128	0	0	0	0	0	0	1

Table S21. Prediction of the susceptible (S), intermediate (I) and resistant (R) categories for Gentamicin. Horizontal axis are the prediction results and the vertical axis are the standard results.

	S	R
S	11	0
R	1	2

Table S22. The standard deviations of the cross-validation results for all metrics.

Standard deviations	RandomForest	SVM-linear	SVM-poly	SVM-rbf	XGboost
GEN	0.06228802	0.054462651	0.069026227	0.064711515	0.135912125
CLI	0.013730124	0.013030835	0.010511181	0.012516067	0.012749381
FOX	0.011481861	0.01099493	0.008669264	0.004532148	0.01170522
LVX	0.024002193	0.019085247	0.013536105	0.014150167	0.016465626
OXA	0.015153003	0.008711123	0.017741377	0.011714621	0.011502216
SXT	0.010206163	0.013701921	0.012932478	0.014155947	0.010586167
DAP	0.03132095	0.023709486	0.028429403	0.02617834	0.009830654
ERY	0.01402437	0.010574013	0.011686653	0.012118157	0.011688437
LNZ	0.017790223	0.034232984	0.032279173	0.030167889	0.016605326
VAN	0.013867403	0.007561768	0.016961876	0.006613238	0.003048994

Table S23. The average of the cross-validation results for all metrics

Average	RandomForest	SVM-linear	SVM-poly	SVM-rbf	XGboost
GEN	0.877869898	0.837106718	0.827620111	0.80341199	0.703290344
CLI	0.951555851	0.950202014	0.944483364	0.94112268	0.94749321
FOX	0.934017561	0.911017429	0.917366927	0.916706749	0.939636148
LVX	0.868256483	0.866439242	0.859483929	0.859364289	0.907621419
OXA	0.938541549	0.924534574	0.911020965	0.906214633	0.946964124
SXT	0.913862482	0.907847002	0.907606509	0.907469758	0.9130294

DAP	0.818540538	0.817347074	0.818277403	0.790125292	0.683269476
ERY	0.926195782	0.924507727	0.909358041	0.903195705	0.940524677
LNZ	0.771851894	0.808850158	0.800079221	0.793496303	0.771304889
VAN	0.944693583	0.951059586	0.939416874	0.948513185	0.952863287

Table S24. Minimum inhibitory concentration (MIC) breakpoints of 10 antimicrobial agents.

Antimicrobial agents	MIC breakpoints (μg/mL)	
Clindamycin	0.5	4
Cefoxitin	4	8
Oxacillin	2	4
Levofloxacin	1	4
Trimethoprim-Sulfamethoxazole	2	4
Vancomycin	2	16
Linezolid	4	8
Erythromycin	0.5	8
Daptomycin	1	2
Gentamicin	4	16

Figure S1. The ROC curves (receiver operating characteristic curve) and the AUC (Area Under Curve) value for Clindamycin.

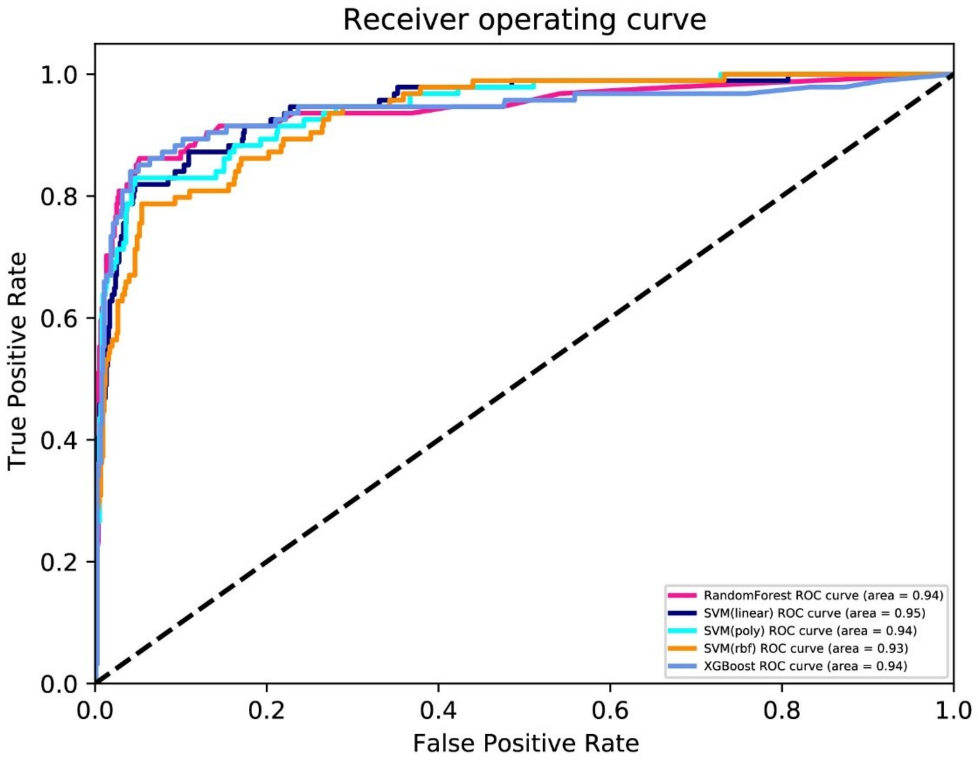

Figure S2. The ROC curves (receiver operating characteristic curve) and the AUC (Area Under Curve) value for Cefoxitin.

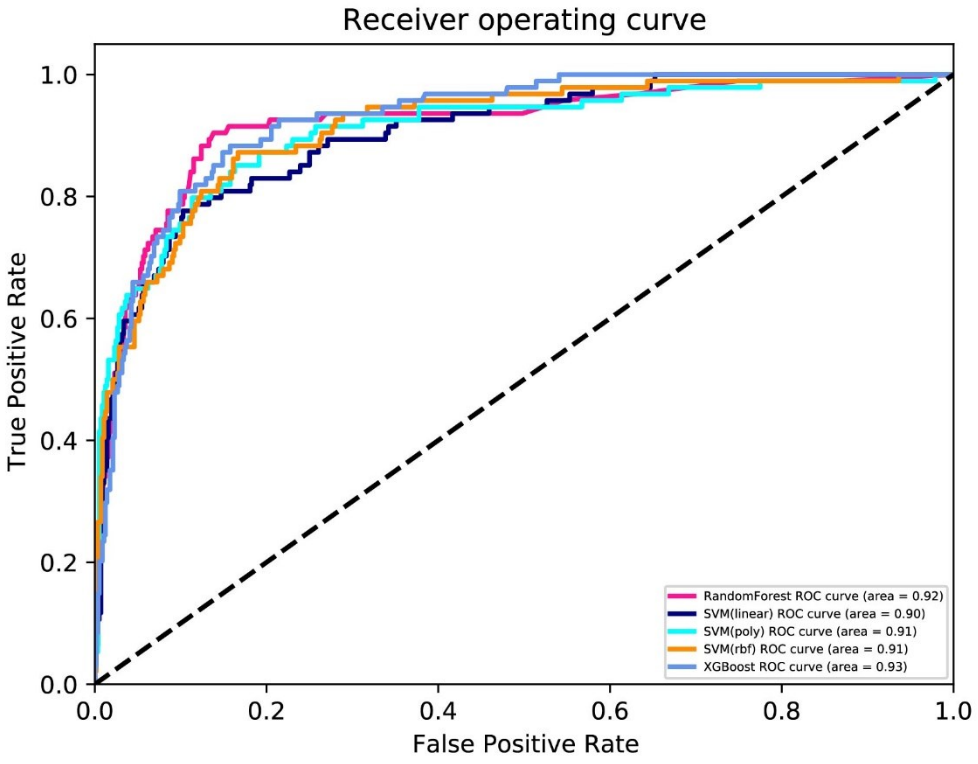

Figure S3. The ROC curves (receiver operating characteristic curve) and the AUC (Area Under Curve) value for Oxacillin.

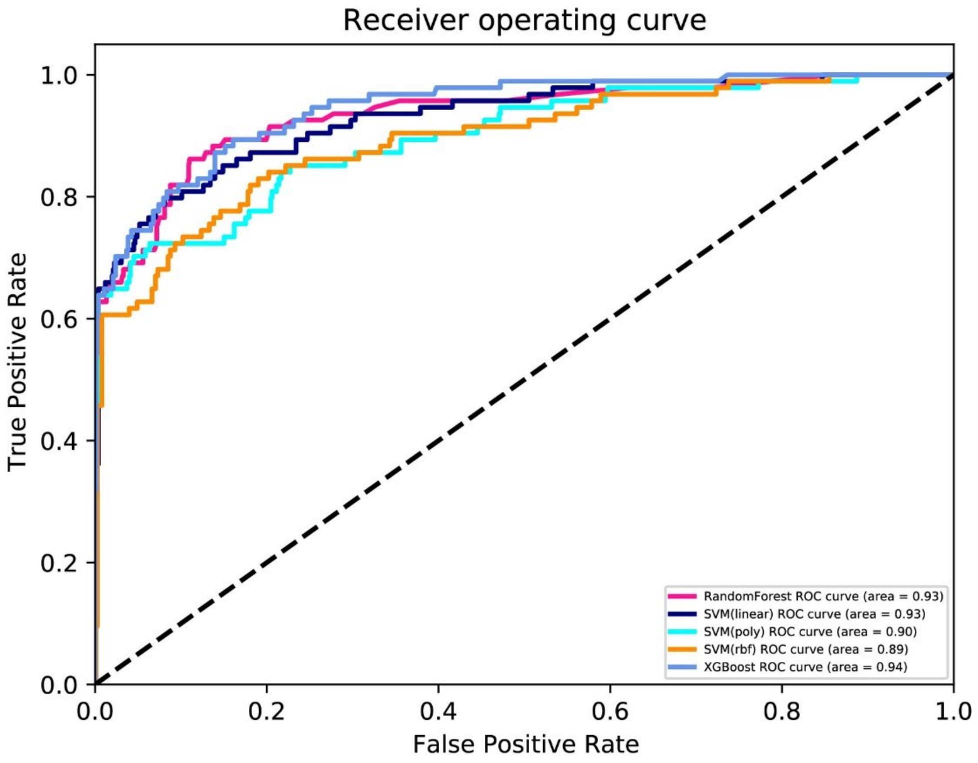

Figure S4. The ROC curves (receiver operating characteristic curve) and the AUC (Area Under Curve) value for Levofloxacin.

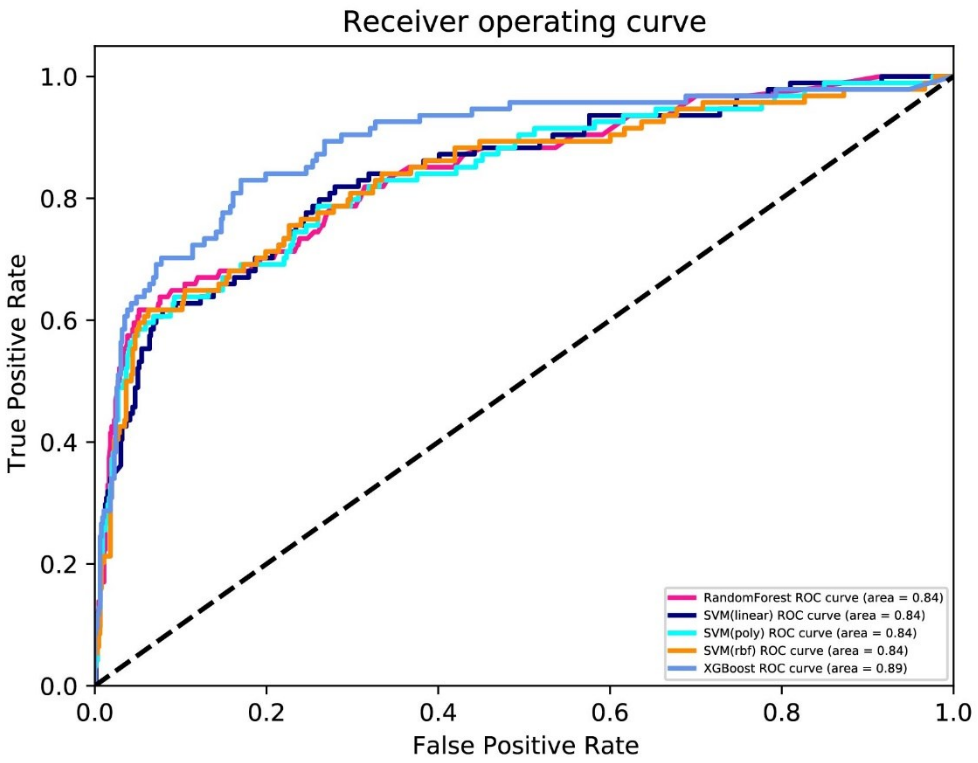

Figure S5. The ROC curves (receiver operating characteristic curve) and the AUC (Area Under Curve) value for Trimethoprim-Sulfamethoxazole.

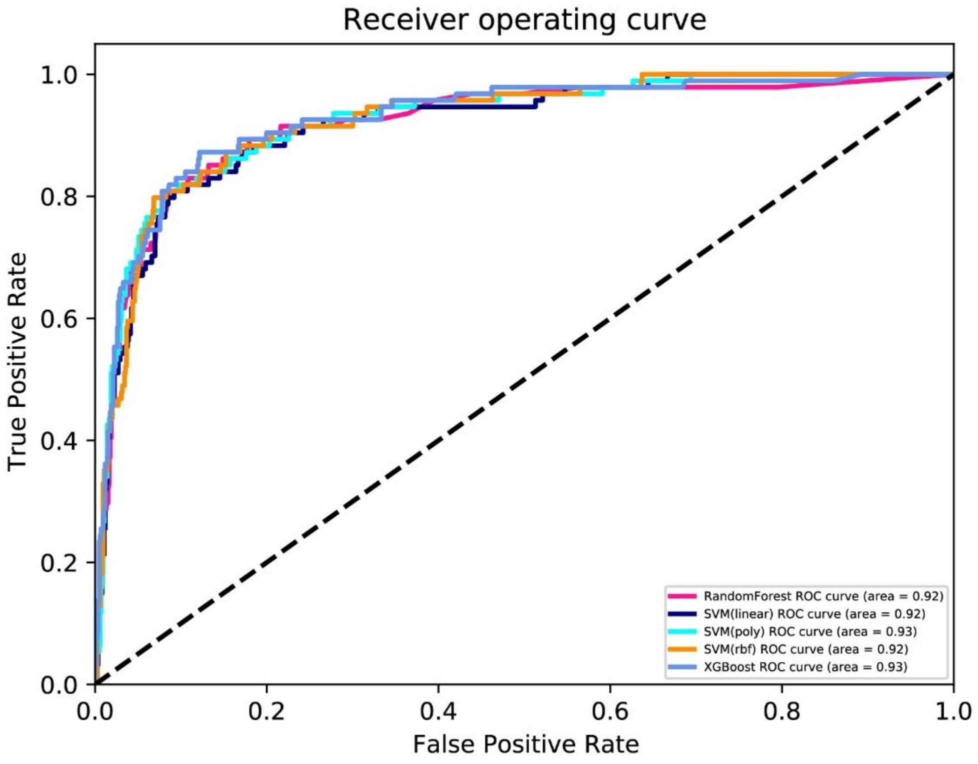

Figure S6. The ROC curves (receiver operating characteristic curve) and the AUC (Area Under Curve) value for Vancomycin.

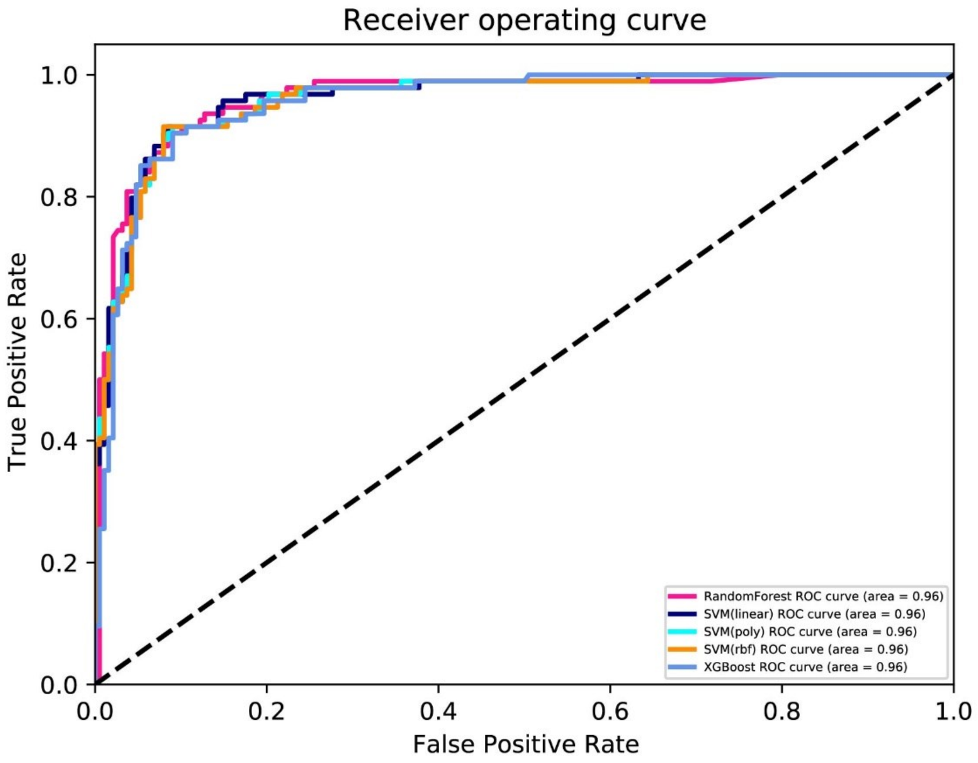

Figure S7. The ROC curves (receiver operating characteristic curve) and the AUC (Area Under Curve) value for Linezolid.

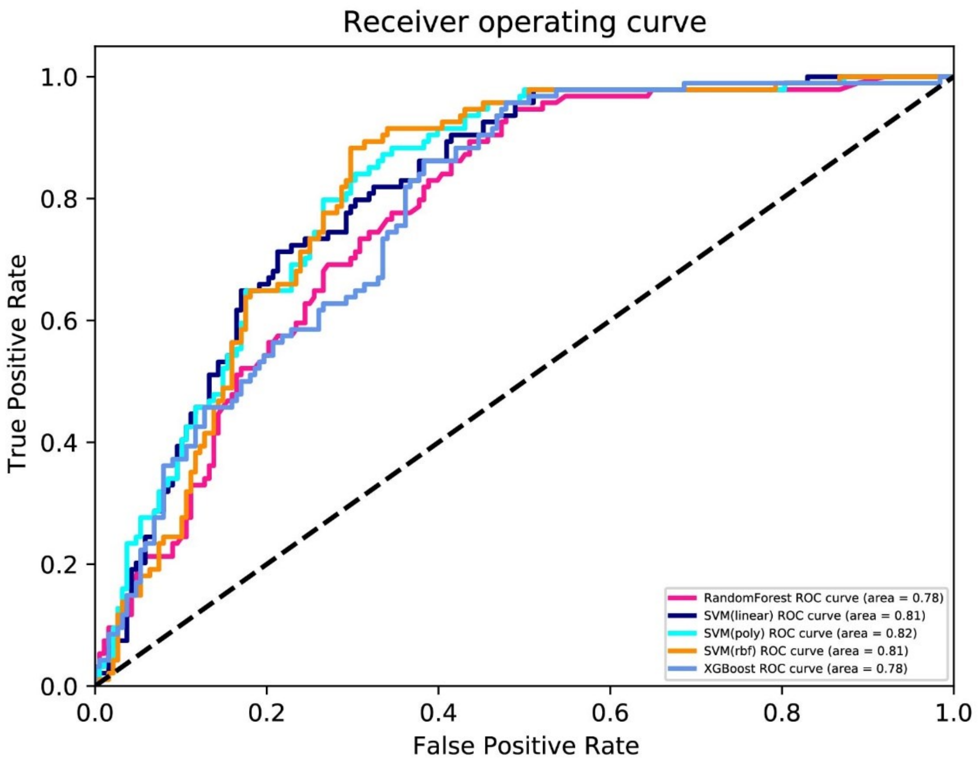

Figure S8. The ROC curves (receiver operating characteristic curve) and the AUC (Area Under Curve) value for Erythromycin.

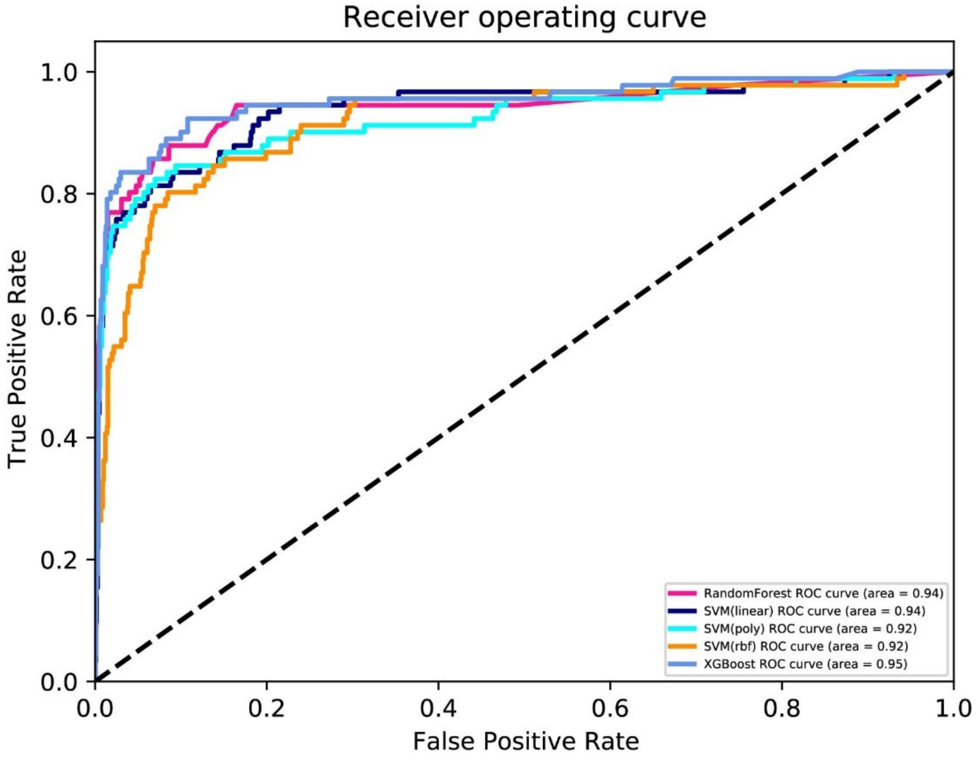

Figure S9. The ROC curves (receiver operating characteristic curve) and the AUC (Area Under Curve) value for Daptomycin.

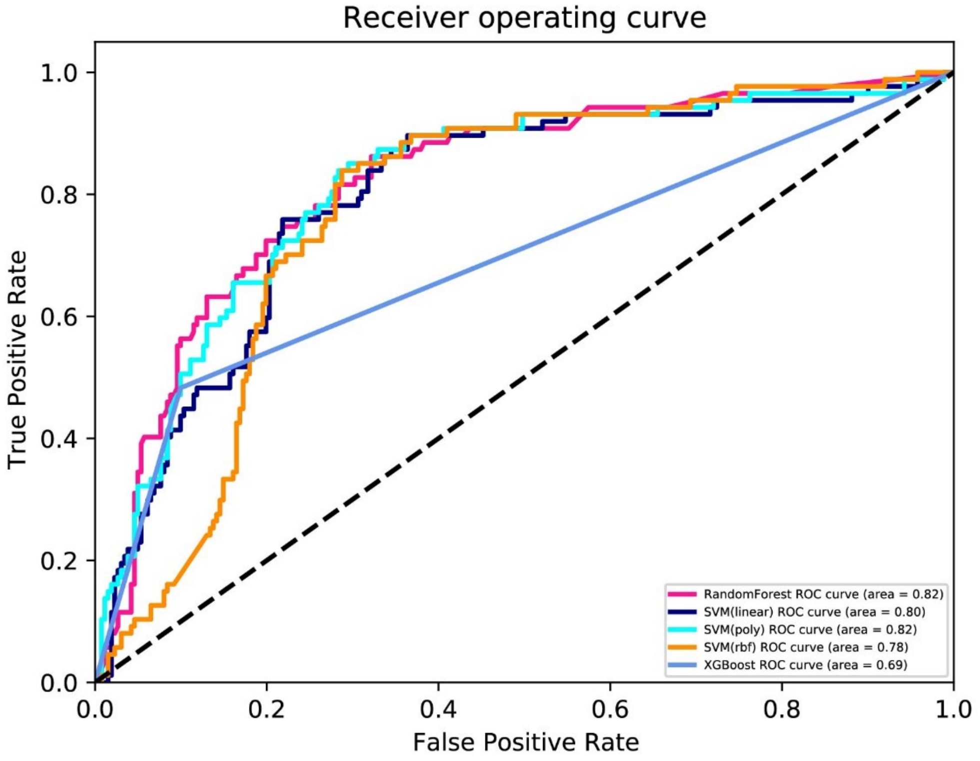

Figure S10. The ROC curves (receiver operating characteristic curve) and the AUC (Area Under Curve) value for Gentamicin.

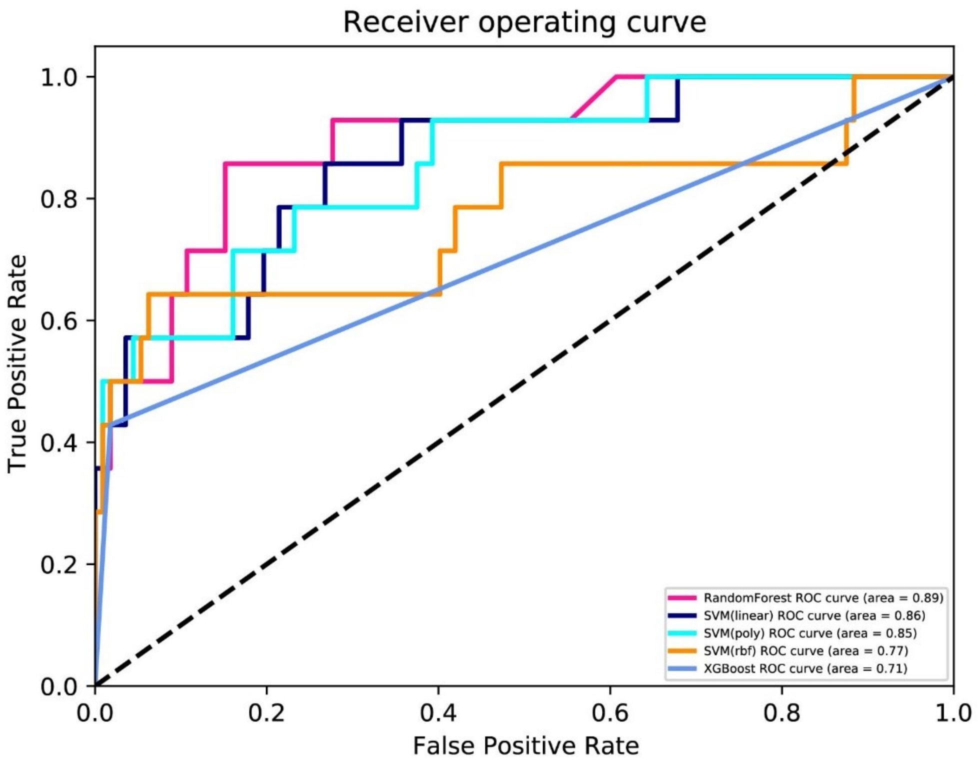

Supplement: Supplementary file 4 [file Data_Sheet_1.PDF]
